# Maternal Separation Alters Ethanol Drinking and Reversal Learning Processes in Adolescent Rats: The Impact of Sex and Glycine Transporter Type 1 (GlyT1) Inhibitor

**DOI:** 10.3390/ijms23105350

**Published:** 2022-05-11

**Authors:** Joanna Filarowska-Jurko, Lukasz Komsta, Irena Smaga, Paulina Surowka, Marta Marszalek-Grabska, Pawel Grochecki, Dorota Nizio, Malgorzata Filip, Jolanta H. Kotlinska

**Affiliations:** 1 Department of Pharmacology and Pharmacodynamics, Medical University, Chodzki 4A, 20-093 Lublin, Poland; joanna.filarowska@gmail.com (J.F.-J.); pawelgrochecki@umlub.pl (P.G.); 2 Department of Medicinal Chemistry, Medical University, Jaczewskiego 4, 20-090 Lublin, Poland; lukasz.komsta@umlub.pl; 3 Department of Drug Addiction Pharmacology, Maj Institute of Pharmacology, Polish Academy of Sciences, Smetna 12, 31-324 Krakow, Poland; smaga@if-pan.krakow.pl (I.S.); surowka@if-pan.krakow.pl (P.S.); mal.fil@if-pan.krakow.pl (M.F.); 4 Department of Experimental and Clinical Pharmacology, Medical University, Jaczewskiego 8b, 20-090 Lublin, Poland; marta.marszalek-grabska@umlub.pl; 5 Experimental Medicine Center, Medical University, Jaczewskiego 8, 20-090 Lublin, Poland; dorotanizio@umlub.pl

**Keywords:** maternal separation, male/female, ethanol drinking, reversal learning, NMDA receptor subunits, GlyT1 inhibitor

## Abstract

Adverse early life experiences are associated with an enhanced risk for mental and physical health problems, including substance abuse. Despite clinical evidence, the mechanisms underlying these relationships are not fully understood. Maternal separation (MS) is a commonly used animal model of early neglect. The aim of the current study is to determine whether the N-methyl-D-aspartate receptor (NMDAR)/glycine sites are involved in vulnerability to alcohol consumption (two-bottle choice paradigm) and reversal learning deficits (Barnes maze task) in adolescent rats subjected to the MS procedure and whether these effects are sex dependent. By using ELISA, we evaluated MS-induced changes in the NMDAR subunits (GluN1, GluN2A, GluN2B) expression, especially in the glycine-binding subunit, GluN1, in the prefrontal cortex (PFC) and ventral striatum (vSTR) of male/female rats. Next, we investigated whether Org 24598, a glycine transporter 1 (GlyT1) inhibitor, was able to modify ethanol drinking in adolescent and adult male/female rats with prior MS experience and reversal learning in the Barnes maze task. Our findings revealed that adolescent MS female rats consumed more alcohol which may be associated with a substantial increase in GluN1 subunit of NMDAR in the PFC and vSTR. Org 24598 decreased ethanol intake in both sexes with a more pronounced decrease in ethanol consumption in adolescent female rats. Furthermore, MS showed deficits in reversal learning in both sexes**.** Org 24598 ameliorated reversal learning deficits, and this effect was reversed by the NMDAR/glycine site inhibitor, L-701,324. Collectively, our results suggest that NMDAR/glycine sites might be targeted in the treatment of alcohol abuse in adolescents with early MS, especially females.

## 1. Introduction

Alcohol use disorders (AUD) are chronic, relapsing disorders characterized by compulsive alcohol intake, loss of control of its consumption, and the presence of negative emotional states during abstinence [1]. Certain factors seem to be associated with increased vulnerability in developing these disorders, such as the early onset of drinking, genetic predisposition, and environmental factors [2,3,4]. Among the deleterious environmental factors compelling AUD development are adverse early life experiences that persist until adulthood [5,6].

In rats, maternal separation (MS), which deprives pups of their mothers, has often been used as a model for adverse early life experiences [7,8]. MS involves the daily separation (15 min to 6 h) of litters from the dams during the first 2–3 weeks of life. These brief separations cause profound neurochemical and behavioral changes in the pups that are found in adulthood, including ethanol intake [9,10] and disruption of learning and memory [11,12,13].

The N-methyl-D-aspartate glutamate receptor (NMDAR) plays an essential role in neuronal development, synaptic plasticity, and learning and memory processes [14]. It is also a major brain target of ethanol. Ethanol potently inhibits NMDAR, and prolonged ethanol exposure leads to compensatory “upregulation” of NMDAR-mediated functions [15]. Studies with recombinant NMDAR have indicated that receptors containing NR1/2A or NR1/2B subunits are generally more sensitive to ethanol inhibition than NR1/2C or NR1/2D receptors [16,17,18]. Of note, NMDAR plays a role in ethanol-induced excitotoxicity and ethanol-induced disturbances of synaptic plasticity [19,20]. Published data indicate that MS with early weaning induces changes in many transmitter systems in the brain, including glutamate and its receptors [21,22,23,24,25,26].

Glycine is an inhibitory neurotransmitter for glycine receptors (GlyR) in the nervous system (CNS). It is also one of the obligatory coagonists of NMDAR (NMDAR/glycine binding site). Glycine binds to the strychnine-insensitive site on the GluN1 subunit of the NMDAR, permitting subsequent binding of glutamate [27,28,29]. Two glycine transporters, GlyT1 and GlyT2, have been identified as important regulators of glycine homeostasis in the brain [30,31]. The expression pattern in the brain of GlyT1, a high-affinity glycine transporter, mirrors the distribution of NMDAR [32]. Persistent hypofunction of GlyT1 [33,34] or acute treatment with potent GlyT1 antagonists, such as sarcosine and NFPS, has been shown to enhance NMDAR currents and long-term potentiation (LTP) in the hippocampus [35,36]. Hence, glycine transporters have been suggested as novel therapeutic targets for brain disorders involving NMDAR dysfunction, such as alcohol dependence [37]. Furthermore, Org 24598, a sarcosine-based and highly selective inhibitor of GlyT1 with a negligible action at GlyT2 and other transporters and receptors [38], shows the ability to reduce the alcohol preference in ethanol-treated animals [39].

Reversal learning measures cognitive flexibility, which is defined as the ability to rapidly adjust established patterns of behavior according to changing circumstances [40]. It has been considered a suitable model for measuring cognitive rigidity in various neuropsychiatric disorders, among others, drug dependence [41,42]. Research indicates that adverse life experiences, such as social isolation or MS can disrupt the reversal learning performance of rats [11,43,44]. Indeed, even a single 24 h MS at postnatal day 8 reduces reversal learning in the Morris water maze (MWM) task of adult CD1 mice [45]. Interestingly, similar results were shown in young adult Wistar rats after single maternal deprivation (24 h) on postnatal day (PND) 9 [46]. Successful reversal learning performance depends on normal prefrontal cortico-striatal functioning, which involves the prefrontal cortex (PFC) [47] and nucleus accumbens (NAc), a major component of the ventral striatum (vSTR) [48].

The PFC, one of the functionally most advanced brain areas, continues to mature after birth through proliferation and migration of neurons, growth of dendrites, and formation of neuronal circuits [49,50]. Furthermore, prolonged postnatal development highlights PFC as a cortical structure particularly sensitive to adverse early life experiences [51,52]. According to in vivo and post-mortem human studies, adverse early life experiences can be associated with functional and structural abnormalities such as impaired cognitive flexibility [53,54], increased impulsivity [55], and reduced PFC cortical density [56,57].

The aim of the current study is to determine whether the NMDAR/glycine sites are involved in vulnerability to alcohol consumption and reversal learning deficits in adolescent rats subjected to the MS procedure and whether these effects are sex dependent. By using ELISA, we evaluated MS-induced changes in the NMDAR subunits (GluN1, GluN2A, GluN2B) expression, especially in the glycine-binding subunit, GluN1, in the PFC and vSTR of male/female rats. Next, we investigated whether Org 24598, a GlyT1 inhibitor, was able to modify ethanol drinking in adolescent and adult male/female rats with prior MS experience in the two-bottle choice paradigms. Finally, we tried to determine whether Org 24598 has an impact on the reversal learning procedure impaired by MS in adolescent rats, using the Barnes maze task. To indicate the involvement of the NMDAR/glycine binding site in the effect of Org 24598 in the reversal learning procedure, we used L-701,324, a glycine site antagonist.

## 2. Results

### 2.1. Effect of Org 24598 on Ethanol Intake in Two-Bottle Choice Paradigm in Male and Female Rats with Prior Maternal Separation

#### 2.1.1. Days 12–14 (Baseline Intake, PND40-42)

**Figure 1:**
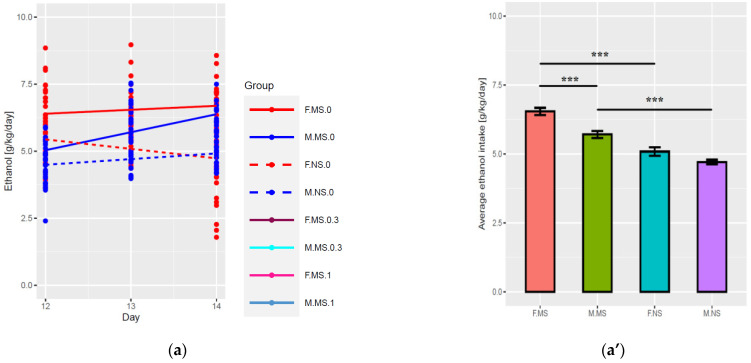

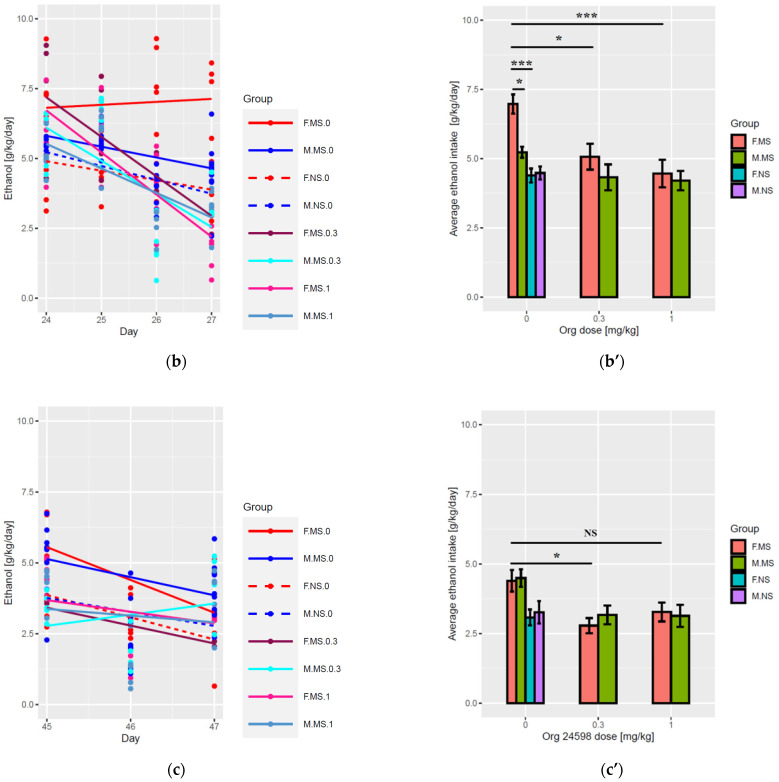
Effect of Org 24598 on ethanol (6% *v*/*v*) intake in two-bottle choice paradigm in male and female Wistar rats (*n* = 8) subjected to MS for 180 min during PND1-21. (**a**,**a’**) Effect of MS on baseline ethanol intake during 12–14th days of drinking procedure (PND40-42); (**b**,**b’**) Effect of Org 24598 (0.3 mg/kg and 1.0 mg/kg) or vehicle treatment on ethanol intake during 24–27th days of drinking procedure (PND52-55) after 1 week of ethanol deprivation; (**c**,**c’**) Effect of Org 24598 (0.3 mg/kg and 1.0 mg/kg) treatment or vehicle on ethanol intake during over days 45–47th drinking procedure (PND73-75) after the following 2 weeks of ethanol deprivation; Left and right panels show the same results, in linear and bar plots. For statistics, see the Results section. PND—postnatal day; MS—maternal separation; NS—non-separated; F—female; M—male; 0—vehicle; 0.3—Org 24598 at the dose of 0.3 mg/kg; 1—Org 24598 at the dose of 1.0 mg/kg. The bar plots show the average ethanol intake over three days of ethanol drinking. * *p* < 0.05; *** *p* < 0.001 vs. non-separated; NS—nonsignificant.

There was a significant difference between males and females (PND40-42) rats in ethanol (6% *v*/*v*) consumption during this period (understood as the difference between linear model intercepts, F(1,244) = 25, *p* < 0.001): females rats consumed more and the average difference was 0.84 ± 0.17 g/kg/day. Furthermore, a significant difference between separated and non-separated animals was observed (F(1,244) = 100, *p* < 0.001): the amount of ethanol consumed by non-separated animals was lower by 1.5 ± 0.17 g/kg/day. The overall changes of slope (the change of consumption in time) between groups (interaction) were significant (F(4,244) = 6.9, *p* < 0.001). There was no significant change of consumption in time for separated females and non-separated males. Significance was observed for: separated males where consumption increased by 0.67 ± 0.15 g/kg/day, (t(244) = 4.36, *p* < 0.001) and non-separated females, where consumption decreased by 0.35 ± 0.15 g/kg/day (t(244) = −2.41, *p* < 0.05) (Figure 1a,a’). Post-hoc analysis with Tukey test showed significant differences (*p* < 0.001) between separated and non-separated animals of both sexes, as well as between sex of separated animals.

#### 2.1.2. Days 24–27 (PND52-55)

There were significant differences between groups in the average ethanol consumption during these days (F(2,155) = 5.1, *p* < 0.01), as well as in the increase or decrease of the consumption in time (F(2,155) = 25, *p* < 0.001). The Org 24598 administration significantly decreased the average ethanol consumption, both for males by 1.1 ± 0.39 g/kg per mg/kg, t(155) = −2.97 (*p* < 0.01) and females per 0.71 ± 0.39 g/kg per mg/kg, t(155) = −1.83 (*p* < 0.1). The Org 24598 administration also increased the slope (the consumption decreased more quickly per day, F(2,155) = 25, *p* < 0.001). The slope change was 1.8 ± 0.3 g/kg/ per mg/kg for females (t(155) = −5.91, *p* < 0.001) and 1.1 ± 0.3 g/kg per mg/kg for males (t(155) = −3.77, *p* < 0.001) (Figure 1b,b’). Post-hoc analysis with Tukey test showed significant differences between separated males and females without Org 24598 administration (*p* < 0.05), as well as between separated and non-separated females (*p* < 0.001). Org 24598 administration caused significant differences only for females, the effect was dose-dependent (*p* < 0.05 for 0.3 mg/kg, *p* < 0.001 for 1 mg/kg).

#### 2.1.3. Days 45–47(PND73-75)

During these days, there were no significant differences in the average ethanol consumptions between the groups (F(3,112) = 0.62, *p* > 0.1), however, there was a significant impact of Org 24598 administration on the ethanol consumption (interaction F(2,112) = 3.8, *p* < 0.05). There were no impact of Org 24598 administration on the slope of ethanol consumption (F(2,112) = 0.87, *p* > 0.1) and no group had a significant change of consumption in time (Figure 1c,c’). Post-hoc Tukey test showed only significant difference (*p* < 0.05) between separated females with 0.3 mg/kg of Org 24598 and without Org 24598.

### 2.2. Effect of Org 24598 on Water Intake in Two-Bottle Choice Paradigm in Male and Female Rats with Prior Maternal Separation

#### 2.2.1. Days 24–27 (PND52-55)

There were overall significant differences in the mean water intake among the four groups (F(3,152) = 3, *p* < 0.05), however, a significant difference in the linear model between MS males and NS males existed at the 90% level (this difference is equal to 9.5 ± 5.5, t(152) = 1.72 (*p* < 0.1). Org 24598 administration did not influence the average water intake in this period (F(2,152) = 0.69, *p* > 0.1), but lowered the slope (intake decreased for subsequent days, F(2,152) = 6.8, *p* < 0.01)). In the linear model, after MS according to the gender, it was significant for males and was equal to 12 ± 3.4 per day per mg/kg (t(152) = −3.65, *p* < 0.001), whereas for females it was equal to 1.9 ± 3.4 and was insignificant (t(152) = −0.57, *p* > 0.1) (Figure S1a,a’). Post-hoc Tukey test did not show any significant differences.

#### 2.2.2. Days 45–47 (PND73-75)

There were overall significant differences in the mean water intake among the four groups (F(3,112) = 6.5, *p* < 0.001). In the linear modeling, significant difference existed only between MS males and NS females—the latter consumed an amount lowered by 18 ± 6.4 t(112) = −2.74, (*p* < 0.01). No significant influence of Org 24598 on the average intake (F(2,112) = 0.74, *p* > 0.1) and slope (F(2,112) = 1.1, *p* > 0.1) was observed (Figure S1b,b’). Post-hoc Tukey test did not show any significant differences.

### 2.3. The Influence of MS on the Spatial Acquisition in the Barnes Maze Task

#### 2.3.1. Latency (PND30-33)

No differences between males and females were observed during this phase (F(1,249) = 0.21, *p* > 0.1). Separation was a significant factor (F(1,249) = 120, *p* < 0.001) and non-separated animals exhibited latency averages that were lowered by −17 ± 7.2. There was a significant negative change of the latency in time (F(1,249) = 130, *p* < 0.001), averaging −3.5 ± 0.59 per day. No significant influence of sex (F(1,249) = 0.21, *p* > 0.1), nor separation (F(1,249) = 2, *p* > 0.1) on this change was observed. There were significant interactions between sex and separation (F(1,249) = 5.4, *p* < 0.05): non-separated males exhibited latency lowered by 3.5 ± 1.5 as compared to separated females (Figure S2a,a’). Post-hoc Tukey test showed significant differences between separated and non-separated group for both sexes (*p* < 0.001), however, there were no significant differences between sex, both for separated and non-separated groups.

#### 2.3.2. Errors (PND30-33)

No differences between males and females were observed during this phase (F(1,249) = 0.99, *p* > 0.1). Separation was a significant factor (F(1,249) = 52, *p* < 0.001) and non-separated animals exhibited error numbers averages that were lowered by −1.5 ± 0.29. There was a significant negative change of the error number in time (F(1,249) = 100, *p* < 0.001), averaging 0.78 ± 0.16 per day. No significant influence of sex (F(1,249) = 0.075, *p* > 0.1), nor separation (F(1,249) = 1.6, *p* > 0.1) on this change was observed. Furthermore, there were no interactions between sex and separation (F(1,249) = 0.024, *p* > 0.1; Figure S2b,b’). Post-hoc Tukey test showed significant differences between separated and non-separated animals, both for males (*p* < 0.05) and females (*p* < 0.01). No significant differences were observed between sexes both for separated and non-separated animals.

### 2.4. Effect of MS on the Probe Trial of the Barnes Maze Task

#### 2.4.1. Latency (PND34)

There were significant differences between separated and non-separated animals (F(1,60) = 59, *p* < 0.001). Non-separated animals exhibited latency averages that were lower by 8.6 ± 1.8. No differences between males and females were observed (F(1,60) = 0.6, *p* > 0.1), as well as no interactions between sex and separation (F(1,60) = 0.68, *p* > 0.1; Figure S3a). Post-hoc Tukey test showed significant differences between separated and non-separated group for both sexes (*p* < 0.001), however, there were no significant differences between sex, both for separated and non-separated groups.

#### 2.4.2. Errors (PND34)

There were significant differences between separated and non-separated animals (F(1,60) = 22, *p* < 0.001). Non-separated animals performed less errors by 2 ± 0.58. No differences between males and females were observed (F(1,60) = 0.37, *p* > 0.1), as well as no interactions between sex and separation (F(1,60) = 0.023, *p* > 0.1; Figure S3b). Post-hoc Tukey test showed significant differences between separated and non-separated group for both sexes (*p* < 0.01), however, there were no significant differences between sex, both for separated and non-separated groups.

### 2.5. Effect of Org 24598 on Reversal Learning of the Barnes Maze in Male/Female Rats with Prior Maternal Separation. Impact of L-701,324

#### 2.5.1. Latency (PND35-37)

There were significant differences between sex in overall mean value of latency (F(1,278) = 10, *p* < 0.01), as well as in the slope (change in time, F(1,281) = 9.7, *p* < 0.01). For males, the mean latency value was 3.7 ± 1.2 s (s) lower than for females and the slope was higher by 4.5 ± 1.4 s per day. Additionally in all cases, the latency value significantly decreased in time (F(1,281) = 250, *p* < 0.001), with an average change equal to −9 ± 1 s per day, t(281) = −8.93, (*p* < 0.001). A significant effect (slope) of Org 24598 (F(2,281) = 25, *p* < 0.001) on the latency was observed. This was significantly lowered for non L-701,324 treated animals by −6.1 ± 1.9 s per mg/kg of Org 24598 dose, t(281) = −3.28, (*p* < 0.01). The Org 24598 effect was slightly higher (3.6 ± 2.2 s) for separated animals, but the difference between Org 24598 effect for separated and non-separated animals was insignificant (F(1,281) = 2.6, *p* > 0.1).

**Figure 2:**
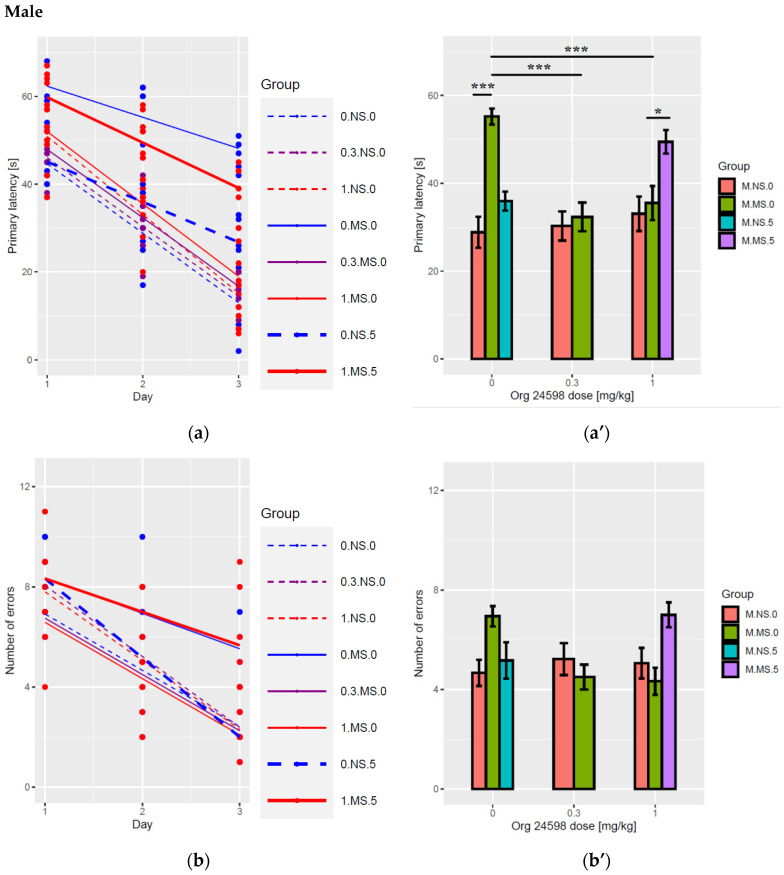

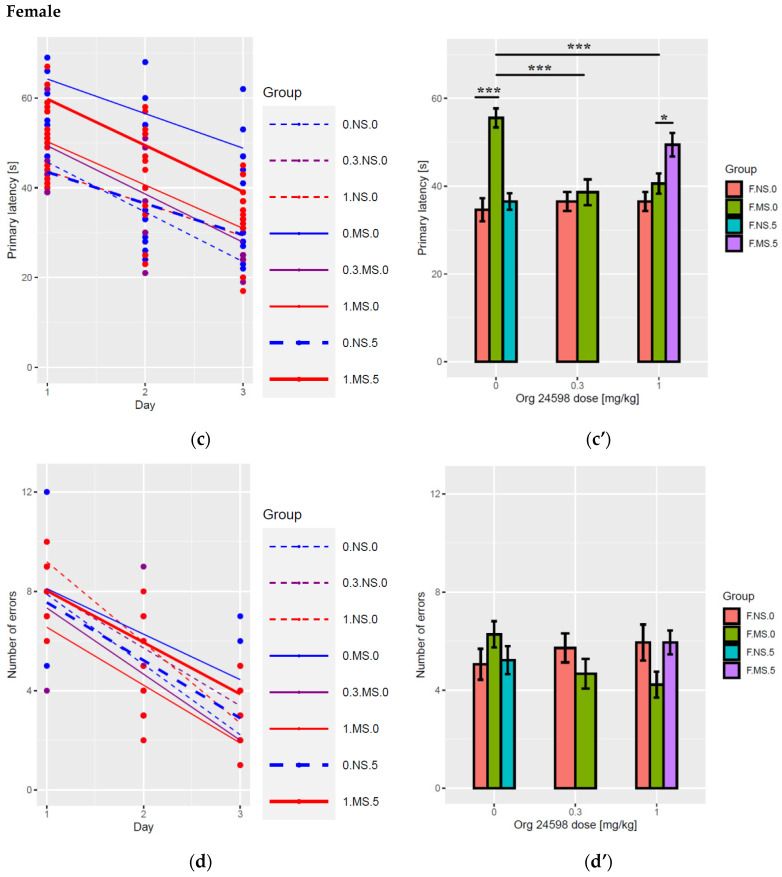
Effect of Org 24598 (0.3 mg/kg and 1.0 mg/kg) or vehicle on the primary latency for males (**a**,**a’**) and females (**c**,**c’**) and number of errors for males (**b**,**b’**) and females (**d**,**d’**) committed during 3 days of the reversal learning phase of the Barnes maze task in adolescent (PND35-37) Wistar rats (*n* = 6) subject to MS for 180 min during PND1-21. Left and right panels show the same results, in linear and bar plots. For statistics, see the Results section. PND—postnatal day; MS—maternal separation; NS—non-separated; 0.3—Org 24598 at the dose of 0.3 mg/kg; 1—Org 24598 at the dose of 1.0 mg/kg; 5—L-701,324 at the dose of 5 mg/kg. The bar plots show the average primary latency and number of errors committed over three days of the reversal learning phase of the Barnes maze. * *p* < 0.05; *** *p* < 0.001 vs. non-separated.

Administration of L-701,324 at the dose of 5 mg/kg reversed the effect of Org 24598 for L-701,324-treated animals (F(2,281) = 25, *p* < 0.001), the effect of Org 24598 equals to 6.9 ± 3 (opposite and significant change, t(281) = 2.15, *p* < 0.05) for L-701,324 treatment (Figure 2a,a’,c,c’). Post-hoc test showed the same significant differences for both sexes: between separated and non-separated group (*p* < 0.001), as well as between non-Org 24598 group and both doses of this substance (*p* < 0.001). For 1 mg/kg Org 24598 dose, administration of L-701,324 caused also significant difference (*p* < 0.05).

#### 2.5.2. Errors (PND35-37)

In all cases, the number of errors significantly decreased in time (F(1,281) = 460, *p* < 0.001), with an average change equal to −2.46 ± 0.16 per day, t(281) = −15.8, (*p* < 0.001). A significant dose-dependent effect of Org 24598 (F(2,281) = 14, *p* < 0.001) on the error number was observed. This was significantly lowered for non L-701,324-treated animals by −2.4 ± 0.45 per mg/kg of Org 24598 dose, t(281) = 5.36, (*p* < 0.001). The Org 24598 effect was lower (1.4 ± 0.34) for separated animals (t(281) = −3.97, *p* < 0.001).

Administration of L-701,324 completely reversed the effect of Org 24598 (1 mg/kg) for L-701,324-treated animals (F(2,281) = 14, *p* < 0.001). The effect of Org 24598 equals to 0.055 ± 0.29 for L-701,324-treatment and does not differ significantly from zero, t(281) = 0.191, (*p* > 0.1). There were no differences in this dataset between sexes (F(1,281) = 0.013, *p* > 0.1) for the main effect (Figure 2b,b’,d,d’). Post-hoc Tukey test did not show any significant differences between groups.

### 2.6. The Influence of MS on Locomotor Activity

Two-way ANOVA with repeated measures did not indicate a significant effect of maternal separation (F(1,28) = 0.005, *p* > 0.05), sex effect (F(1,28) = 0.32, *p* > 0.05) and maternal separation × sex interaction (F(1,28) = 0.09, *p* > 0.05) on the locomotor activity in rats after the probe trial. All data are shown in Figure S4.

### 2.7. Biochemical Analysis

#### The Influence of Early Maternal Separation on the Expression of NMDAR Subunit Proteins, Namely GluN1, GluN2A and GluN2B in the PFC and vSTR 24 h after the Last Separation Procedure in Male and Female Rats

**Figure 3:**
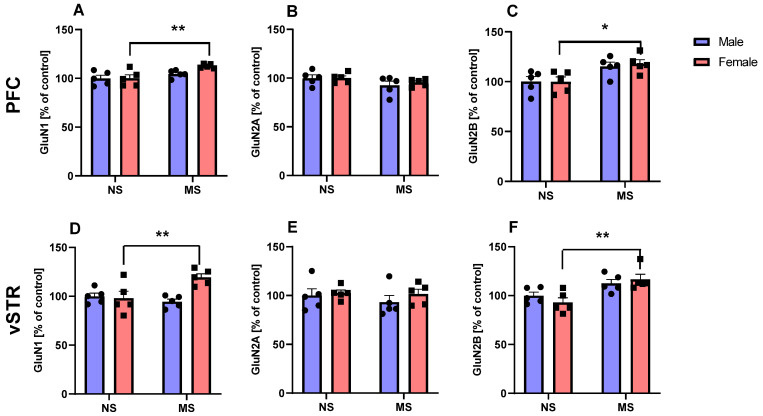
Effect of MS on GluN1 (**A**,**D**), GluN2A (**B**,**E**), and GluN2B (**C**,**F**) subunits of NMDAR in the PFC and vSTR of adolescent male and female rats (PND22) subjected (N = 5/group) to MS for 180 min during PND1-21. The concentration of proteins was calculated from a standard curve and presented as a percentage of control. PND—postnatal day; MS—maternal separation; NS—non-separated; PFC—prefrontal cortex; vSTR—ventral striatum. * *p* < 0.05; ** *p* < 0.01 vs. non-separated.

Two-way ANOVA with repeated measures indicated a significant effect of maternal separation on the GluN1 in the PFC (F(1,16) = 10.79, *p* < 0.01) but no sex effect (F(1,16) = 2.418, *p* > 0.05) and maternal separation × sex interaction (F(1,16) = 2.418, *p* > 0.05). Bonferroni post-hoc test showed that maternal separation significantly increased GluN1 in the PFC of females in comparison to non-separated rats (*p* < 0.01; Figure 3A). Two-way ANOVA with repeated measures did not indicate a significant effect of maternal separation (F(1,16) = 3.97, *p* > 0.05), sex effect (F(1,16) = 0.72, *p* > 0.05) and maternal separation × sex interaction (F(1,16) = 0.23, *p* > 0.05) on the GluN2A in the PFC (Figure 3B). Instead, two-way ANOVA with repeated measures indicated a significant effect of maternal separation on the GluN2B in the PFC (F(1,16) = 13.38, *p* < 0.01) but no sex effect (F(1,16) = 0.08, *p* > 0.05) and maternal separation x sex interaction (F(1,16) = 0.08, *p* > 0.05; Figure 3C). Bonferroni post-hoc test showed that maternal separation significantly increased GluN2B in the PFC of female in comparison to non-separated rats (*p* < 0.05; Figure 3C).

Two-way ANOVA with repeated measures indicated a significant effect of sex effect (F(1,16) = 6.48, *p* < 0.05) and maternal separation × sex interaction (F(1,16) = 8.86, *p* < 0.01) but no maternal separation (F(1,16) =3.11, *p* > 0.05) on the GluN1 in the vSTR (Figure 3D). Bonferroni post-hoc test showed that maternal separation significantly increased GluN1 in the vSTR of females in comparison to non-separated rats (*p* < 0.01; Figure 3D). Two-way ANOVA with repeated measures did not indicate a significant effect of maternal separation (F(1,16) = 0.47, *p* > 0.05), sex effect (F(1,16) = 0.94, *p* > 0.05) and maternal separation × sex interaction (F(1,16) = 0.25, *p* > 0.05) on the GluN2A in the vSRT (Figure 3E). Instead, two-way ANOVA with repeated measures indicated a significant effect of maternal separation on the GluN2B in the vSTR (F(1,16) = 16.28, *p* < 0.001) but no sex effect (F(1,16) = 0.10, *p* > 0.05) and maternal separation × sex interaction (F(1,16) = 1.54, *p* > 0.05; Figure 3F). Bonferroni post-hoc test showed that maternal separation significantly increased GluN2B in the vSRT of females in comparison to non-separated rats (*p* < 0.01; Figure 3F).

## 3. Discussion

The present study demonstrates that MS induced a higher vulnerability to develop alcohol drinking in the two-bottle choice paradigm in adolescent rats, with sex differences in overall ethanol intake. The female MS group consumed more ethanol than their male counterparts. Furthermore, the MS procedure induced significant upregulation of the GluN1 subunit of NMDAR in the PFC and vSTR only in female rats, suggesting hypofunction of glycine signaling. Still, in both sexes, MS induced significant upregulation of the GluN2B subunit of the NMDAR in these brain structures. Org 24598, a GlyT1 inhibitor, decreased the ethanol drinking in adolescent rats with prior MS, with a significant linear response between dose and effect. MS also induced deficits in reversal learning in adolescent rats in the Barnes maze task, with similar effects in both sexes. Org 24598 ameliorated deficit in reversal learning, and this effect of Org 24598 was abolished by L-701,324—an antagonist of the NMDAR/glycine site. As far as we know, this is the first study investigating molecular changes associated with the NMDAR/glycine site in MS-induced neuroplasticity in adolescent rats.

### 3.1. NMDAR/Glycine Site and Vulnerability to Ethanol Drinking in MS Male/Female Rats

Traumatic early life events may increase the risk of developing AUD, and MS is a risk factor for alcohol consumption in later life [58]. Preclinical research has shown that MS resulted in faster behavioral sensitization to ethanol [59,60] and increased vulnerability to ethanol consumption [61], mainly in female mice. Interestingly, the literature shows inconsistencies over this issue [4,59,62,63,64,65,66]. Our study supports and extends the previous work in mice and indicates that MS increases ethanol intake in the two-bottle choice paradigm in adolescent rats (PND40-42). Furthermore, the female MS group was much more vulnerable to alcohol consumption than the males. For females, ethanol consumption was stable over time, but males increased daily ethanol consumption across days of drinking experience (baseline intake). Thus, the present evidence supports and extends those observations that implicate sex differences [61,67] in MS-induced ethanol intake.

In addition, the current study revealed that MS young adult (PND52-55) rats continuously consumed more ethanol than NS animals after one week of ethanol deprivation. Still, female rats drank more ethanol than males. The female ethanol drinking was stable during voluntary consumption, but male ethanol drinking decreased over time. Previous experiments indicated that GlyT1 inhibitors such as Org 25935 and Org 24598 in a dose-dependent manner reduced ethanol intake and preference in male Wistar rats [68,69,70] with chronic ethanol consumption. Our study confirms that Org 24598 declined the ethanol consumption in both sexes in MS rats, although its impact on ethanol consumption was more pronounced in female rats and appears to be dose-dependent, in contrast to males, where it appears not to be dose-dependent.

Interestingly, an increase in ethanol consumption in the MS group of rats was not observed after the second withdrawal period (procedure day 45–47), when animals were adult (PND73-75). However, they continuously drank more than the NS rats. Org 24598 still decreased the ethanol consumption in both sexes from the MS group, significantly in female rats. This effect is difficult to explain and needs further investigation. It should be noted that Org 24598 did not have an influence on ethanol consumption in the NS animals, suggesting that the Org 24598 effect was selective for the MS rats. Furthermore, this finding suggests that tolerance to the inhibitory effect of Org 24598 on ethanol drinking did not develop over time. On the other hand, during our study, in general, there were no significant differences in water consumption due to Org 24598 administration. Org 24598 decreased the consumed amount of water over time only for separated males during drinking days 24–27. In addition, the same group indicated large reductions in ethanol consumption. This phenomenon is difficult to explain because such differences were not observed during the last phase (procedure day 45–48) of the two-bottle choice paradigm.

The present study showed that MS in neonatal rats upregulates the GluN1 subunit of NMDAR (due to glycine signaling hypofunction) in the rat PFC, and such effect was significant in adolescent females. In other studies, mRNA levels for NMDAR proteins were evaluated and the results showed a significantly increased level of NR1 mRNA in the PFC of female offspring [26]. Beyond the aforementioned, an upregulation of GluN2B subunit in the PFC of both sexes was observed. Altogether, these findings suggest that MS can cause NMDAR dysfunction (hyperglutamatergic state) in the PFC of female adolescent rats. In adolescent male rats, in contrast, MS induced only a slight increase in the GluN1 subunit. Of note, other researchers have revealed that chronic exposure to ethanol is accompanied by an increase in GluN1 and GluN2B levels both in vivo and in vitro, and it is widely believed that these adaptive changes play an important role in the development of alcohol dependence and in the withdrawal syndrome [71]. Therefore, glycine transporter inhibition and NMDA/glycine site agonist reduce the hyperglutamatergic state associated with alcoholism [39,72,73,74].

Preclinical data indicated that deficient recruitment of NMDAR coagonists such as glycine or serine in the ventromedial PFC (vmPFC) may underlie the enhanced impulsivity observed during protracted alcohol abstinence [75]. Rats with long-term ethanol exposure demonstrated improper response inhibition in the five-choice serial reaction time task (5-CSRTT) [75,76,77,78] associated with diminished recruitment of the vmPFC glycine and serine. Administration of glycine inhibitor (ALX5407) into the vmPFC alleviated deficits in impulse control through a mechanism reliant on the availability of the glycine site on the NMDAR. The attenuation of premature responding in ethanol rats following intra-vmPFC ALX5407 administration was fully blocked by coadministration of the selective NMDAR/glycine site antagonist L-701,324 [79]. Furthermore, persons with alcohol disorders who are abstinent also exhibit dysregulation of glycine signaling that contributes to increased impulsivity during protracted alcohol abstinence [75]. Together, these results demonstrate a functional link between deficient NMDA glycine coagonist signaling and increased impulsivity during protracted ethanol abstinence. Thus, a hypofunctioning state of glycine signaling at the NMDA coagonist site in the PFC of MS female rats may lead to a constraint of motor impulsivity. Given that prolonged MS produces an increase in impulsivity in adolescence [80] and given that impulsivity is predictive of compulsive drug-taking, we hypothesize that impulsivity could be a predictor of the ethanol vulnerability that was presented in our study in female MS rats.

On the other hand, the GluN1 subunit of the NMDA receptor interacts with the dopamine D1 receptor (D1R), and in the presence of the GluN2B subunit, such D1R/GluN1 complexes in the STR [81] integrate dopamine and glutamate signaling to control synaptic plasticity and behavior in response to drug abuse. Herein, D1R promotes drug reinforcement, and it may increase the motivational effects of ethanol [82]. It seems that female adolescent rats with early life MS procedures have lower levels of glycine than male rats, therefore they are more sensitive to the rewarding and motivational effects of ethanol. Ethanol intake and preference decreased after systemic administration of Org 24598, a GlyT1 inhibitor. However, a limitation of our study is that we did not use L-701,324 to support the involvement of the NMDAR/glycine sites in the drinking procedure. Still, our study in the reversal learning task confirmed that NMDAR/glycine sites are affected by Org 24598. Collectively, these results give further support to the concept of elevating central glycine levels to reduce ethanol intake in MS rats.

### 3.2. NMDAR/Glycine Site and Reversal Learning in MS Male/Female Rats

The present data regarding reversal learning in the Barnes maze task revealed that MS rats tested at adolescence (PND35-37) showed less flexibility in learning to reverse a previously learned association compared to NS littermates. MS rats required more trials to reach the criterion and made more errors than their NS littermates during the reversal phase of the task. Moreover, our study shows that males and females needed nearly the same time to reach the shelter, suggesting no significant sex differences in reversal learning trials in the Barnes maze task. Thus, these data indicate that MS induces changes in cognitive flexibility, an important domain of executive function, early in life when rats have just been weaned. Previously, Wang et al. [11] showed that MS induced decreased reversal learning with age in the Morris water maze (MWM) with no significant sex differences. Other research reported reversal learning deficits in the MWM of adult CD1 mice with a single 24 h MS at PND8 [45] or in young adult Wistar male rats with a single 24 h MS at PND9 in the T-maze test [46]. It is well known that the effects of MS on cognitive function are related to differences in the experimental design, animal strain, age, or other factors. Still, the deficits in reversal learning suggest that subjects with MS have problems with the ability to adapt established patterns of behavior to changing circumstances or rules. Moreover, such changes in chronic ethanol abuse patients have been correlated with a higher incidence of relapse [83]. Published data suggests that these deficits depend critically on the PFC [84,85,86,87].

NMDAR plays a pivotal role in neuronal development, learning, memory, and synaptic plasticity [88]. Antagonists of GlyT1 increase levels of glycine in the synaptic cleft and, like direct glycine site agonists, can augment NMDAR currents and NMDAR-mediated functions such as LTP [35]. Our study shows that Org 24598, a GlyT1 inhibitor, ameliorated MS-induced deficit in reversal learning with no dose-effect. Furthermore, coadministration of the NMDAR/glycine site antagonist—L-701,324 reversed completely the effects of Org 24598 (1 mg/kg), and such effects were seen in male and female rats. Thus, our results confirmed that the blockade of GlyT1 causes an increase in the NMDA function and LTP. Since LTP is a mechanism underlying memory formation, these results suggest that GlyT1 antagonists could enhance learning and memory processes. Therefore, our results demonstrate a functional link between deficient NMDA coagonist signaling and cognitive flexibility impairment induced by MS in rats.

In addition, the GluN2B subunit of NMDAR in the PFC and vSTR circuits was also upregulated in adolescent rats of both sexes. However, published data indicate that only the early phase of reversal learning may depend on Glu2B subunit signaling, but generally, rats learn to navigate the new location over several trials [89], suggesting that alterations in Glu2B subunit signaling only delay learning processes. GlyT-1 inhibitors, can enhance this process [90].

Reversal learning procedures are just one of an array of tasks designed to measure aspects of impulsive actions and choices. During reversal learning, subjects must suppress one response while engaging actively in another to obtain a reward. The reversal learning could index vulnerability for disorders characterized by impulsivity, such as proclivity for initial substance abuse, as well as the compulsive aspects of dependence [41]. As dysregulation of glycine signaling contributes to impulsivity [76], we cannot exclude the involvement of impulsivity in the reversal learning task in our study, especially in females. However, more research is needed to confirm this hypothesis.

In conclusion, our study indicated that MS increased vulnerability to ethanol drinking preferentially in female rats and showed deficits in reversal learning in both sexes. An increased vulnerability to ethanol drinking is associated with a substantial upregulation of the GluN1 subunit of NMDAR, suggesting hypofunction of glycine signaling. Herein, Org 24598, a GlyT1 inhibitor, attenuated ethanol consumption preferentially in female rats and reversed learning deficits in both sexes. Thus, our results suggest that NMDAR/glycine sites might be targeted in the treatment of alcohol abuse in adolescents with early MS, especially females. Published data indicated that even long glycine therapy does not lead to life-threatening side effects [91,92,93].

## 4. Materials and Methods

### 4.1. Animals

The study was approved by the Local Ethics Committee (125/2018) in Lublin under the ‘3R approach’ (Replace, Reduce and Refine) and performed according to the National Institute of Health Guidelines for the Care and Use of Laboratory Animals, as well as the European Community Council Directive of November 2010 for Care and Use of Laboratory Animals (Directive, 2010/63/EU) (IACUC equivalent approval). The subjects used in the present study were offspring of Wistar dams (OMD, Lublin, Poland) mated in the animal facility at the OMD, Lublin, Poland. The dams were housed individually throughout the gestation in polypropylene cages (41 cm × 34 cm × 16 cm) with approximately 3 cm layer of sawdust shavings on the cage floor. Rodent chow (Sniff Spezialdiäten GmbH, Soest, Germany) and water were available ad libitum to the animals throughout the study. The experiments were maintained under standard laboratory conditions (22 ± 1 °C, 12:12 light/dark cycle, lights on at 8:00). All behavioral experiments were performed between 9:00 am and 7:00 pm. The day of birth was designated as PND 0.

### 4.2. Drugs

Org 24598 lithium salt (*N*-Methyl-*N*-[(3*R*)-3-phenyl-3-[4-(trifluoromethyl) phenoxy]propyl]-glycine lithium salt) was purchased from Tocris Bioscience (Bristol, UK) and given at the dose of 0.3 and 1.0 mg/kg. L-701,324 (7-chloro-4-hydroxy-3-(3-phenoxy)phnyl-2-(1H)-quinolone) was purchased from Merck Sharp & Dohme (Natick, MA, USA) and given at the dose of 5 mg/kg. Both substances were dissolved in saline with 0.1% DMSO [94] and given at the volume of 2 mL/kg, intraperitoneally (i.p.). Org 24598 was administered 30 min before the testing session, but L-701,324 was given 30 min before Org 24598 injection.

### 4.3. Maternal Separation

The maternal separation (MS) procedure was carried out from PND1 to PND21. The total number of dams used in the present study was 20 (10 litters for MS stress and 10 litters for control). The procedure of MS was based on the protocol described by Chocyk et al. [95] with minor modifications. On each of PND1–21, the dams and the pups were removed from the maternity cages for 180 min (09:00 to 12:00). The mothers were placed individually in holding cages, while each litter was placed in a cardboard container lined with fresh bedding material. The containers were moved to a bigger cage where adequate ambient temperature (34 °C) was provided by placing hot water bottles on the bottom. After the 180 min separation, the pups and the dams were returned to the maternity cages. Once a week, the maternity cages were cleaned during one of the separation procedures. Control, non-separated (NS) animals were left undisturbed with their mothers, except during the cage cleaning that was performed once a week. During the MS procedure, male and female segregation was not performed. During the weaning period, male and female segregation was performed, animals were then assigned to two independent procedures: (i) two-bottle choice paradigm and (ii) reversal learning of the Barnes maze task.

### 4.4. Two-Bottle Choice Paradigm

Eight days after the last MS procedure, on PND29, the rats (*n* = 8/group) were placed separately in plastic cages (30 cm × 30 cm × 30 cm) and introduced to ethanol in a two-bottle free-choice paradigm. The animals were randomly assigned to drug and vehicle groups in all experiments. Next, the rats were given access to one bottle with water and one bottle with ethanol (95%, POCH, Gliwice, Poland) solution in gradually increasing concentrations (*v*/*v*) from 2% to 6% during a period of 14 days (4 days with 2%, 3 days with 4%, 7 days with 6%). The 6% ethanol (*v*/*v*) solution was used for Wistar rats since ethanol consumption in this strain is highest at this concentration [96]. In all cages, the location of the two bottles was changed every day to prevent side preference. On days 12–14 (PND40-42) of the bottle choice procedure, the baseline intake of ethanol and water was established. At this time, water and ethanol intake were measured daily, and ethanol consumption (g/kg/day) and water consumption (mL/kg/day) were calculated.

After the last day of baseline measurement, the alcohol bottles were removed from the cages, leaving the rats with free access to food and water for 7 days. On the 22nd day of the bottle choice procedure (PND50), animals were again subjected to ethanol (6% *v*/*v*) for 7 days. In addition, each animal was subjected to a total of five injections (starting at 7:00 am on PND50 with 12-h intervals) of either vehicle or Org 24598 (0.3 mg/kg and 1.0 mg/kg, i.p.). The alcohol bottles were reintroduced after the second injections (at 7:00 pm on day 22). Total ethanol (g/kg/day) and water intake (mL/kg/day) were measured daily at 7:00 pm between 24–27 days (PND52-55) of the bottle choice procedure.

**Figure 4:**
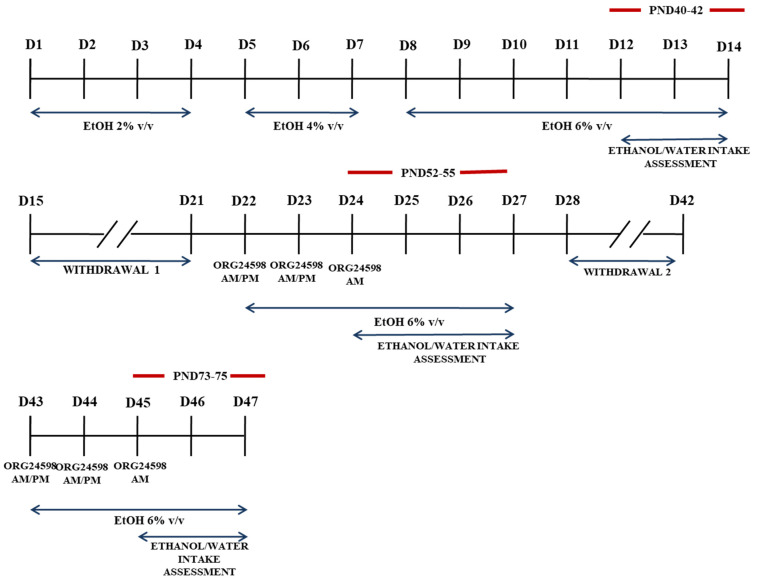
Experimental outline used for voluntary consumption of ethanol in adolescent adult Wistar rats subjected to MS during PND1-21 for 180 min. The timeline shows the sequence and duration of the experimental protocol. EtOH—ethanol; D—day of experiment; AM—morning; PM—afternoon.

Additionally, after 14 days of ethanol withdrawal, on days 43–47 (PND71-75) of the two-bottle choice procedure, animals were again subjected to ethanol (6% *v*/*v*) for 5 days. Herein, similar to the previous procedure, each animal was subjected to a total of five vehicle or Org 24598 (0.3 mg/kg and 1.0 mg/kg, i.p.) injections (starting at 7:00 am on PND73 with 12-h intervals). The alcohol bottles were reintroduced after the second Org 24598 injection (at 7:00 pm on PND73). Total ethanol (g/kg/day) and water intake (mL/kg/day) were measured daily at 7:00 pm between 45–47 days (PND73-75) of the two-bottle choice procedure (Figure 4).

### 4.5. Barnes Maze Task

The Barnes maze task was carried out according to the method described by Kuzmin et al. [97] with minor modifications. The Barnes maze apparatus (Stoelting, Dublin, Ireland) consisted of a circular grey metal platform (diameter 122 cm), elevated 100 cm above the floor, with 20 holes (10 cm diameter) located in the periphery of the platform. One hole was connected to an escape box of 35 cm × 12 cm × 12 cm of the same material and color as the platform. The other holes were covered underneath with a flat box, also of the same material and color so that the rats could not discriminate the escape hole from other holes until situated adjacent to it. In addition, numerous visual cues (in the form of large colorful geometric shapes) were placed on the walls of the testing room at 1–2 m distance from the edge of the maze. To evoke the potentiated escape response, the platform was brightly lit (two points of light 1.5 m above the maze: 500 W each) as an aversive stimulus. The Barnes maze task was run in 4 phases: habituation, acquisition, probe trial, and reversal learning.

#### 4.5.1. Habituation

On PND29, one day before the acquisition phase, to reduce anxiety behavior, the rats were habituated to the platform and the escape box. This habituation trial was performed with the lights on.

#### 4.5.2. Acquisition Phase

One day (24 h) after the maze habituation, the same rats were subjected to the acquisition phase (PND30-33). Acquisition involved one training session per day for 4 consecutive days. Each training session consisted of two 180 s trials, with a 5-min inter-trial interval when the animals were returned to their home cages. The location of the platform and the escape box remained constant over all the acquisition trials. Each trial began by placing the animal at the center of the platform and then it was allowed to freely explore the apparatus. The trial was completed after 180 s or when the animal entered the escape box. Immediately after entering the escape box, the hole was covered for 30 s before the rat was returned to its home cage. If the animal did not enter the goal box within 180 s, it was gently guided there by the experimenter and could explore it for 30 s. To dissipate odor cues and to provide a standard olfactory context for each trial, the platform surface and the escape box were wiped with a 10% (*w*/*v*) ethanol solution after each trial [98]. All trials were recorded by a trained observer. Since the animals occasionally lacked motivation and merely explored the maze after finding the escape box rather than entering into it, following the work of many authors [99,100,101,102], in our experiments, we scored such parameters as the primary latency and primary errors. Primary latency was defined as the time required for the rat to make initial contact with the escape box. Primary errors were defined as the number of holes visited before the first contact with the escape box.

#### 4.5.3. Probe Trial

One day after the acquisition phase (PND34), the subjects received a probe trial for 90 s to evaluate spatial memory. During this trial, the tunnel leading to the escape box was closed. The rats were allowed to explore the maze and investigate the escape box and the adjacent holes. The primary latency and primary errors to reach the escape box were counted.

#### 4.5.4. Reversal Learning

One day after completion of the probe trial, two 180 s reversal learning trials were run for three consecutive days (PND35-37). Reversal learning trials were identical to the acquisition trials, except that the position of the escape hole was rotated 180°. The rat was, therefore, unable to escape the maze using the acquired spatial cues but had to relearn the new location of the hole. Before the first reversal learning trial, the animals were assigned to experimental groups as presented in Table 1. Org 24598 was administered once daily 30 min before every first reversal learning trial for the three days of reversal learning sessions. L-701,324 was administered 30 min before Org 24598 or vehicle injection. Data obtained from the reversal learning trials were pooled together and used for calculations of primary latency and primary errors.

### 4.6. Locomotor Activity

The locomotor activity of individual rats was recorded using a photocell apparatus (Porfex, Bialystok, Poland) immediately after the probe trial. The animals were individually placed in Plexiglas boxes (square cages, 60 cm a side) in a sound-attenuated experimental room. The cages were equipped with two rows of infrared, light-sensitive photocells, located 40 and 100 mm above the floor. Locomotor activity was recorded as a horizontal activity (distance traveled) by each rat for a total period of 15 min.

### 4.7. ELISA Assays

One day after completion of the MS procedure the rats were sacrificed, the brains were dissected and the PFC (PFC; +3.20 to +2.20 mm from bregma) and vSTR (vSTR: +1.2 to ±2.5 mm from bregma) were selected according to the Paxinos and Watson [103] histological atlas for reference. The brain structures were frozen in and stored at −80 °C until the biochemical analysis.

Quantitative measurement of NMDA receptor subunits in rat brain structures was performed using a Rat Glutamate (NMDA) receptor subunit Zeta-1 ELISA Kit (E1005Ra; Bioassay Technology Laboratory, Shanghai, China), a Rat Glutamate (NMDA) receptor subunit epsilon-1 ELISA Kit (E1205Ra; Bioassay Technology Laboratory, China), and a Rat Glutamate (NMDA) receptor subunit epsilon-2 ELISA Kit (E1204Ra; Bioassay Technology Laboratory, Shanghai, China) following manufacturers’ protocols. Briefly, frozen brain structures were homogenized in cold PBS (pH 7.4) containing cocktails of protease and phosphatase inhibitors (Sigma-Aldrich, St. Louis, MO, USA) using a homogenizer ball (Bioprep-24, Allsheng, China) (10 s at 10,000 rpm). Then, homogenates were centrifuged for 5 min at 5000× *g*, the supernates were immediately removed, and protein concentration in the supernates was measured using a bicinchoninic acid assay (BCA) protein assay kit (Serva, Heidelberg, Germany). Subsequently, 100 µg of protein from each sample was used in the ELISA assays. Duplicates of each sample and series of standards were transferred to ELISA plates. The absorbance was measured at a wavelength of λ = 450 nm using a Multiskan Spectrum spectrophotometer (Thermo LabSystems, Philadelphia, PA, USA). The concentration of proteins was calculated from a standard curve and presented as a percentage of control.

### 4.8. Statistical Analysis

All data treatment in the two-bottle choice and reversal learning experiments was conducted in open-source GNU R computational environment version 4.1.1 (www.r-project.org). The obtained results were split according to phases and each phase was fitted to a linear model containing the factors as quantitative or qualitative variables. The day number was treated as a quantitative variable (to investigate the significance of a change in time, i.e., the slope of the equation), as well as Org 24598 dose (to investigate the significance of dose–response correlation). The remaining variables (sex, separation, and the presence of L-701,324) were inserted into the model as qualitative variables to investigate the significance of their impact on intercepts and slopes. The ANOVA test was performed on the models to enhance the statistical analysis with overall significances of interactions.

The multi-way model with one quantitative variable (time) used in the study is almost the same as a two-way ANOVA test with sex and separation as factors. The F results are indeed ANOVA results, removing all other factors from this model’s outcomes with only a slight change of obtained significance. Comparisons between groups were made by applying the Tukey post-hoc test. ELISA data were expressed as means (± SEM). Statistical analyses were performed using two-way ANOVA with Bonferroni post-hoc test. *p* < 0.05 was considered statistically significant.

## Figures and Tables

**Table 1:** Experimental groups used in the reversal learning of the Barnes maze task.

	Substance	Sex	Stress	N
1.	Vehicle + Vehicle	Male/Female	-	6
2.	Vehicle + Org 24598 0.3 mg/kg	Male/Female	-	6
3.	Vehicle + Org 24598 1.0 mg/kg	Male/Female	-	6
4.	L-701,324 5 mg/kg + Vehicle	Male/Female	-	6
5.	Vehicle + Vehicle	Male/Female	+	6
6.	Vehicle + Org 24598 0.3 mg/kg	Male/Female	+	6
7.	Vehicle + Org 24598 1.0 mg/kg	Male/Female	+	6
8.	L-701,324 5 mg/kg + Org 24598 1.0 mg/kg	Male/Female	+	6

## Data Availability

The data presented in this study are available on request from the corresponding author.

## Supplementary Materials

The following supporting information can be downloaded at: https://www.mdpi.com/article/10.3390/ijms23105350/s1.
