# Supplementary material for: Maternal Separation Alters Ethanol Drinking and Reversal Learning Processes in Adolescent Rats: The Impact of Sex and Glycine Transporter Type 1 (GlyT1) Inhibitor
Source: Int J Mol Sci. 2022 May 11;23(10):5350. doi: 10.3390/ijms23105350 (PMC9141364; doi:10.3390/ijms23105350)
Supplement: Supplementary file 1 [file ijms-23-05350-s001.zip › ijms-1697183-supplementary.pdf]

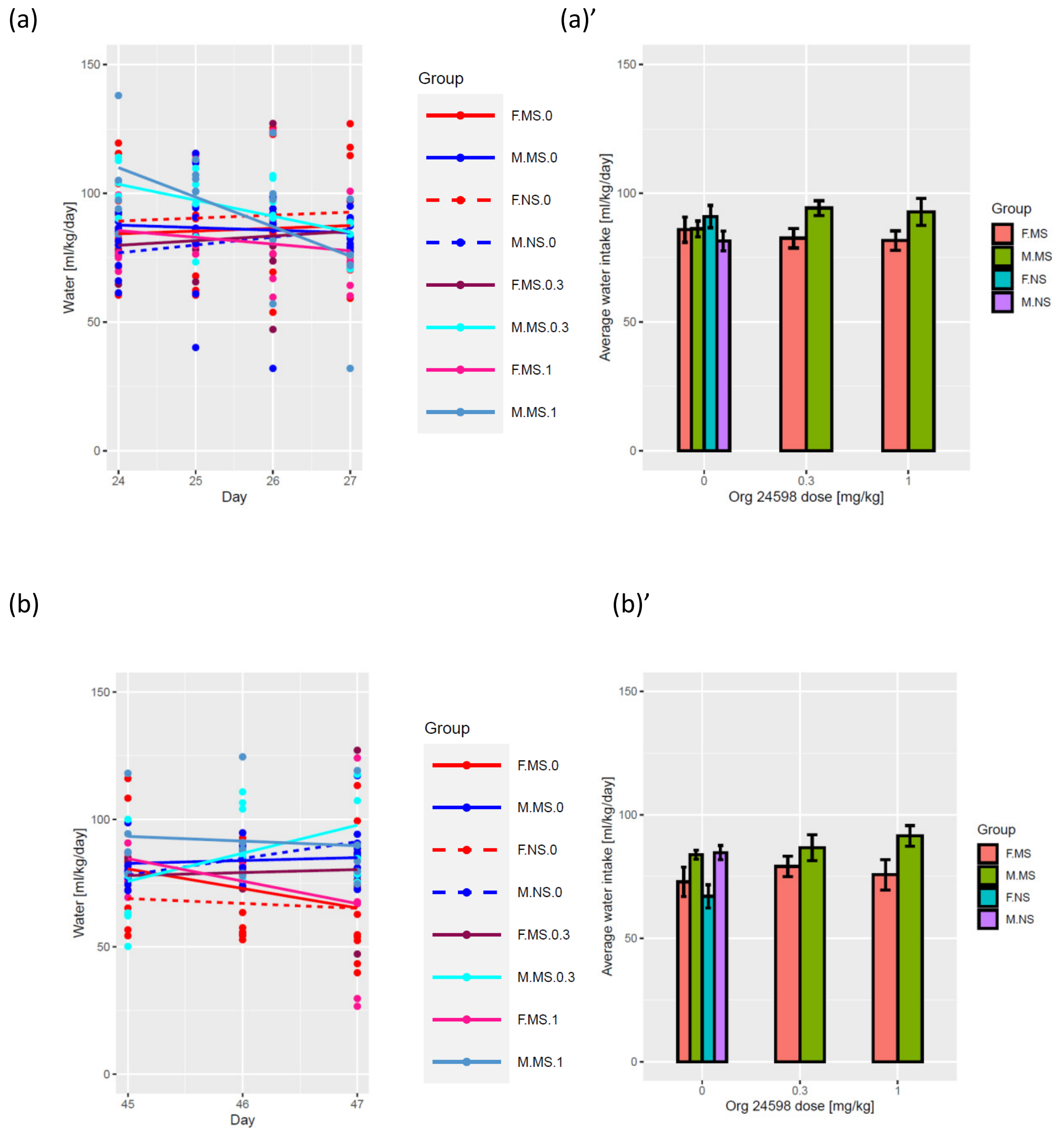

Figure S1. Effect of Org 24598 on water intake in two-bottle choice paradigm in male and female Wistar rats (n=8) subjected to MS for 180 min during PND1-21. (a, a') Effect of Org 24598 (0.3 mg/kg and 1.0 mg/kg) or vehicle treatment on water intake during 12-14th days of drinking procedure (PND52-55); (b, b') Effect of Org 24598 (0.3 mg/kg and 1.0 mg/kg) or vehicle treatment on water intake during PND73-75. Left and right panels show the same results, in linear and bar plots. For statistics, see the Results section. PND – postnatal day; MS - maternal separation; NS – nonseparated; F- female; M -male; 0 – vehicle; 0.3 – Org 24598 at the dose of 0.3 mg/kg; 1 – Org 24598 at the dose of 1.0 mg/kg. The bar plots show the average water intake over three days of water drinking.

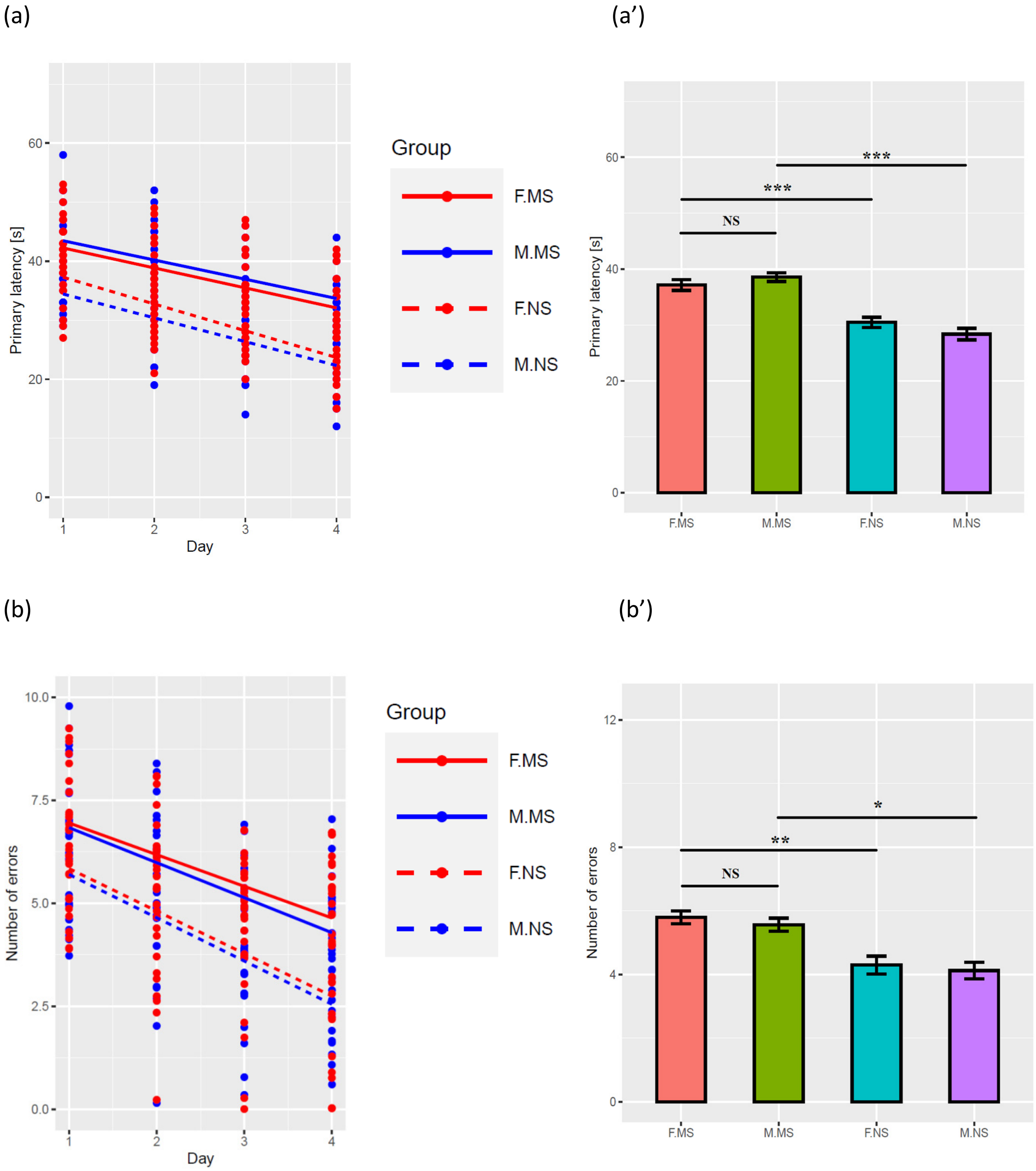

Figure S2. Effect of MS on the primary latency (a, a') and number of errors (b, b') for males and females committed during 4 days of the acquisition phase of the Barnes maze task in adolescent (PND30-33) Wistar rats subject to MS for 180 min during PND1-21. Left and right panels show the same results, in linear and bar plots. For statistics, see the Results section. PND – postnatal day; MS - maternal separation; NS – non-separated; F- female; M –male. The bar plots show the average primary latency and number of errors committed over three days of the acquisition phase of the Barnes maze. * P<0.05; **P<0.01; ***P<0.001 vs non-separated; NS – nonsignificant.

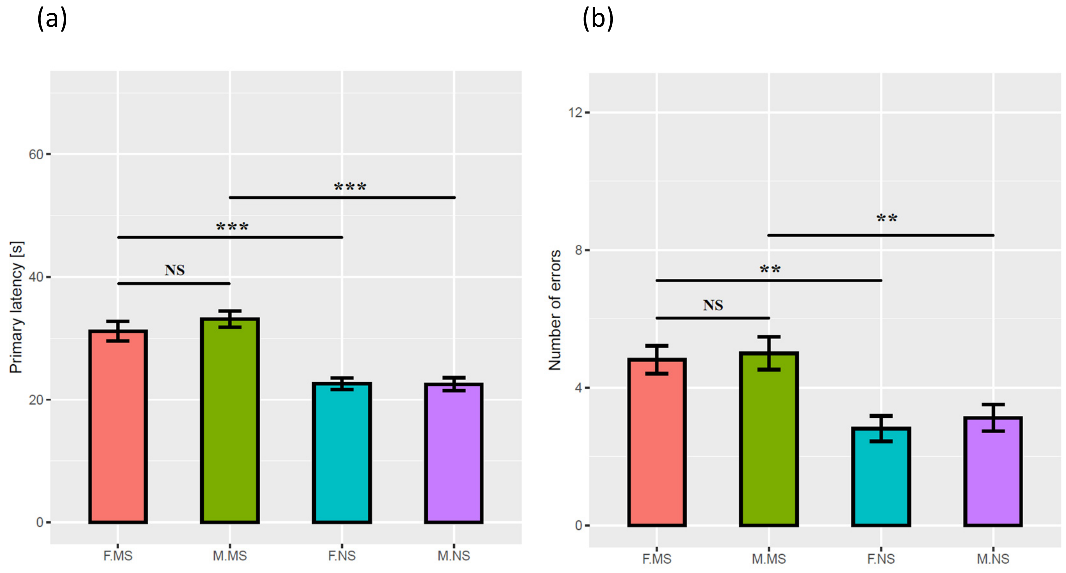

Figure S3. Effect of MS on the primary latency (a) and number of errors (b) committed by male and female Wistar rats on the probe trial day (PND34). For statistics, see the Results section. PND – postnatal day; MS - maternal separation; NS – non-separated; F- female; M –male. ** P<0.01; *** P<0.001 vs non-separated; NS – nonsignificant.

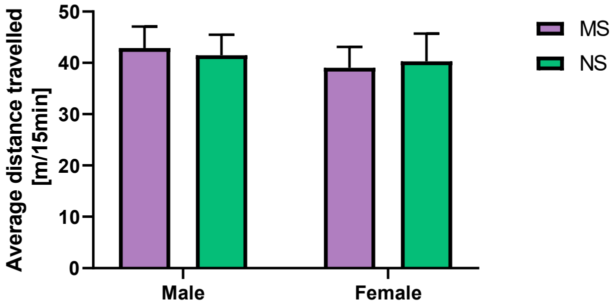

Figure S4. The influence of MS on locomotor activity after the probe trial of the Barnes' maze. For statistics, see the Results section. MS - maternal separation; NS – non-separated.
